# Hypothalamic 2-Arachidonoylglycerol Regulates Multistage Process of High-Fat Diet Preferences

**DOI:** 10.1371/journal.pone.0038609

**Published:** 2012-06-21

**Authors:** Sei Higuchi, Keiichi Irie, Ryuji Yamaguchi, Mai Katsuki, Maiko Araki, Makiko Ohji, Kazuhide Hayakawa, Shohei Mishima, Yoshiharu Akitake, Kiyoshi Matsuyama, Kenji Mishima, Kenichi Mishima, Katsunori Iwasaki, Michihiro Fujiwara

**Affiliations:** 1 Department of Neuropharmacology, Faculty of Pharmaceutical Sciences, Fukuoka University, Fukuoka, Japan; 2 Institute for Aging and Brain Sciences, Fukuoka University, Fukuoka, Japan; 3 Department of Chemical Engineering, Faculty of Engineering, Fukuoka University, Fukuoka, Japan; University of Tübingen, Germany

## Abstract

**Background:**

In this study, we examined alterations in the hypothalamic reward system related to high-fat diet (HFD) preferences. We previously reported that hypothalamic 2-arachidonoylglycerol (2-AG) and glial fibrillary acid protein (GFAP) were increased after conditioning to the rewarding properties of a HFD. Here, we hypothesized that increased 2-AG influences the hypothalamic reward system.

**Methods:**

The conditioned place preference test (CPP test) was used to evaluate HFD preferences. Hypothalamic 2-AG was quantified by gas chromatography-mass spectrometry. The expression of GFAP was examined by immunostaining and western blotting.

**Results:**

Consumption of a HFD over either 3 or 7 days increased HFD preferences and transiently increased hypothalamic 2-AG levels. HFD consumption over 14 days similarly increased HFD preferences but elicited a long-lasting increase in hypothalamic 2-AG and GFAP levels. The cannabinoid 1 receptor antagonist O-2050 reduced preferences for HFDs after 3, 7, or 14 days of HFD consumption and reduced expression of GFAP after 14 days of HFD consumption. The astrocyte metabolic inhibitor Fluorocitrate blocked HFD preferences after 14 days of HFD consumption.

**Conclusions:**

High levels of 2-AG appear to induce HFD preferences, and activate hypothalamic astrocytes via the cannabinoid system. We propose that there may be two distinct stages in the development of HFD preferences. The induction stage involves a transient increase in 2-AG, whereas the maintenance stage involves a long lasting increase in 2-AG levels and activation of astrocytes. Accordingly, hypothalamic 2-AG may influence the development of HFD preferences.

## Introduction

Subjects often choose to consume a high-fat diet (HFD) over other diets; this is called a HFD preference. Many reports have shown that rats and mice exhibit spontaneous HFD preferences [1], [2]. Excessive consumption of HFDs induces life-style related diseases including obesity, diabetes, and hyperlipidemia. The etiology of HFD preferences has not yet been fully elucidated but it is known that HFD preferences often induce excessive consumption of HFDs. Several studies have indicated that food reward, such as the consumption of a HFD, is controlled by the endogenous brain reward system [1], [2], [3], [4], [5]. It is thought that preferences for HFDs are attributable to numerous endogenous factors that are related to the reward system. We focused on the cannabinoid system as a potential mediator of HFD preferences because cannabinoids are involved in physiological processes related to memory, reward-related feeding, energy balance, and related processes [5], [6], [7], [8], [9], [10], [11], [12], [13]. Previously, we reported that the brain levels of the endocannabinoid 2-arachidonoylglycerol (2-AG), which is a full agonist of cannabinoid 1 (CB_1_) receptors, are increased in mice with HFD preferences [14]. Moreover, our data suggested that the development of preferences for HFDs requires the activation of hypothalamic astrocytes via CB_1_ receptors [15]. Therefore, 2-AG may be intimately involved in the relationship between HFD preferences and the brain reward system. From these findings, in this study we hypothesized that 2-AG may influence the hypothalamic reward system and the process of developing a HFD preference.

The cannabinoid system, which includes CB_1_ receptors and 2-AG, has been known to play a major role in the regulation of reward and appetite [5], [6], [7], [8], [10], [11], [12], [13]. Recently, blockade of CB_1_ receptors has been shown to suppress appetite and consumption of attractive diets such as HFDs, sweet food, and alcohol [13], [16], [17], [18], [19], [20], [21], [22]. We reported that hypothalamic 2-AG is increased in the mice with a HFD preference, and the CB_1_ receptors antagonist O-2050 reversed HFD preferences [14]. CB_1_ receptors are found in the hypothalamus [23], [24]. In addition, recent findings indicate that direct infusion of CB_1_ receptor antagonists into the hypothalamus attenuates food consumption and appetite [17]. From these data, we hypothesized that the hypothalamic cannabinoid system plays important roles in HFD preferences. In this study, we used the conditioned place preference test (CPP test) to evaluate preferences for HFDs. The CPP test has been used to evaluate the rewarding effects of addictive drugs and highly palatable diets [1], [25], [26], [27], [28], [29]. The CPP test measures rewarding effects based on the subject's preference for a specific environment that has been previously paired with an appetitive stimulus. In this study, we quantified the levels of hypothalamic 2-AG before and after the CPP test to examine the relationship between the preference for a HFD and hypothalamic 2-AG.

In a previous study, our data suggested that hypothalamic astrocytes may be involved in the development of preferences for HFDs via activation of CB_1_ receptors [15]. Astrocytes are non-neuronal cells in the brain, and they regulate neurotransmission [30], [31], [32]. Furthermore, recent studies show that activated astrocytes are involved in drug abuse [33], [34], [35], [36]. These reports suggest the possibility that astrocytes may play important roles in the reward system. However, there is no direct evidence of a relationship between astrocytes and rewarding effects. To this end, in the present study, we used the astrocyte metabolic inhibitor fluorocitrate (FC) to directly examine the relationship between astrocytes and HFD preferences.

## Results

**Figure 1:**
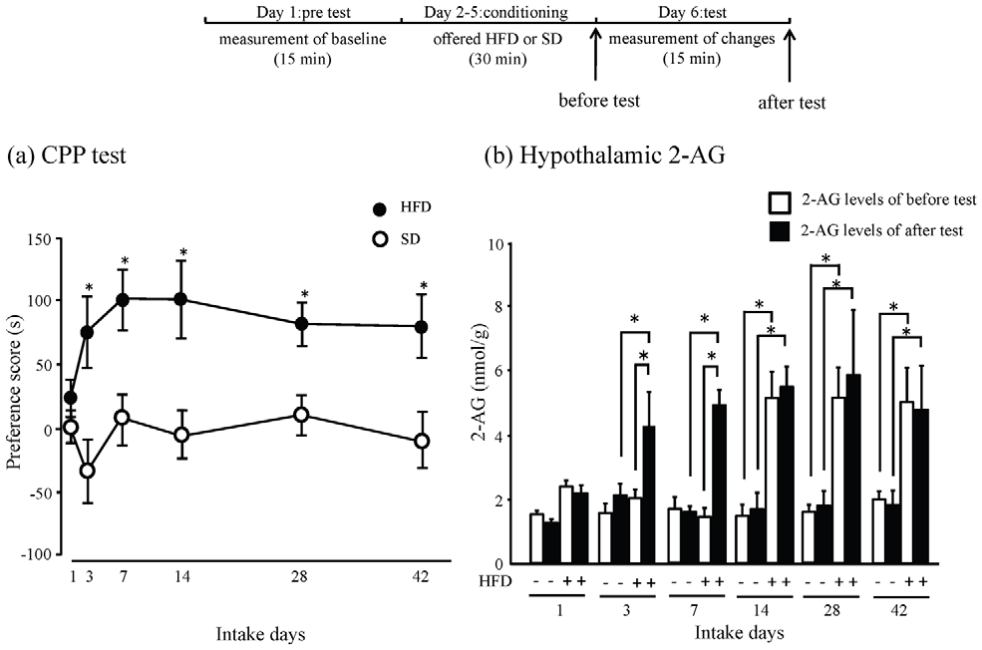
Preference scores and hypothalamic 2-AG after SD or HFD consumption. (a) Filled circles represent the preference score in HFD intake mice. Open circles represent the preference score in SD intake mice. Preference score represents the mean change in time (s) spent in the HFD-paired side in pre-test and test sessions. n = 8 for each. Results are expressed as the mean ± S.E.M. *p<0.05 vs. SD consumption group (Student's t-test were performed following two-way ANOVA). (b) Mice were given HFD (+) or SD (-) before the CPP test. Hypothalamic 2-AG were quantified by GC-MS. Filled squares represents the hypothalamic 2-AG after test. Open squares represents the hypothalamic 2-AG before test. n = 8 for each. Results are expressed as the mean ± S.E.M.

**Figure 2:**
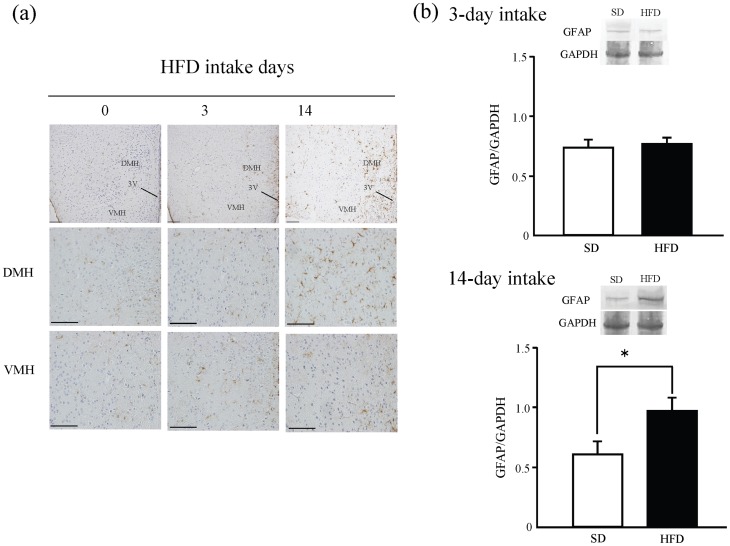
The expression of GFAP in the hypothalamus after HFD consumption. (a) Immunostaining of GFAP after 0, 3, or 14 days of HFD intake in the hypothalamus Abbreviations: 3 V: 3rd ventricle; DMH: dorsomedial hypothalamic nucleus; VMH: ventromedial hypothalamic nucleus. Scale bar: 100 μm. (b) The expression of GFAP after 3 or 14-day HFD intake. n = 6 for each. Results are expressed as the mean ± S.E.M. ^*^ *p*<0.05 vs. SD (Student's t-test).

**Figure 3:**
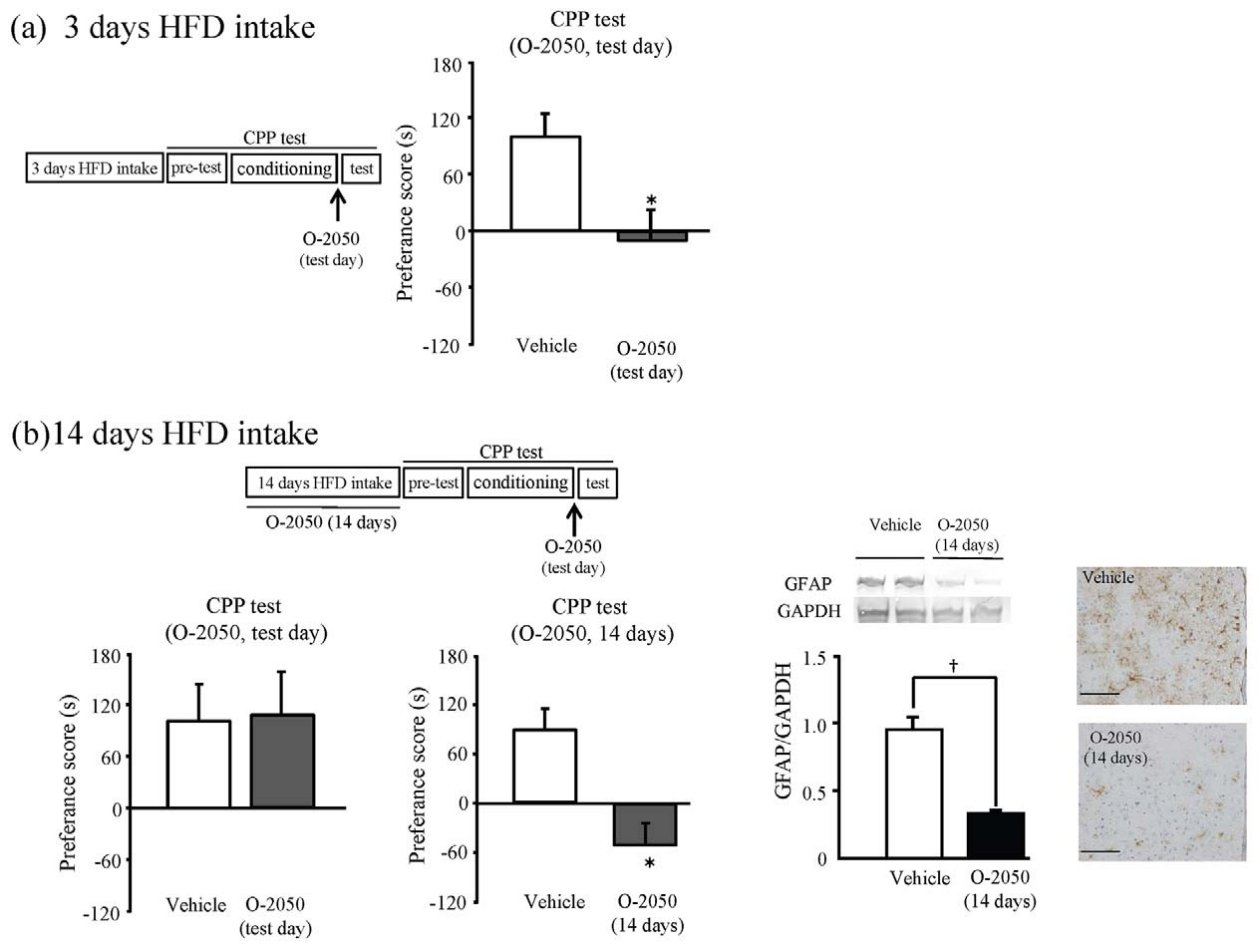
Effects of the CB_1_ receptor antagonist O-2050 on HFD preferences. (a) Mice were given HFD for 3 days before CPP test. O-2050 (10 mg/kg i.p.) was administrated 1 h before the test. n = 8 for each. Results are expressed as the mean ± S.E.M. ^*^ *p*<0.05 vs. Vehicle (Student's t-test). (b) Mice were given HFD for 14 days before CPP test. O-2050 (10 mg/kg i.p.) was administrated 1h before the test (test day) or for 14 days before the CPP test (14 days). n = 8 for each (CPP test). n = 6 for each (western blotting). Results are expressed as the mean ± S.E.M. Scale bar: 100 μm. ^*^ *p*<0.05 vs. Vehicle (Student's t-test).

**Figure 4:**
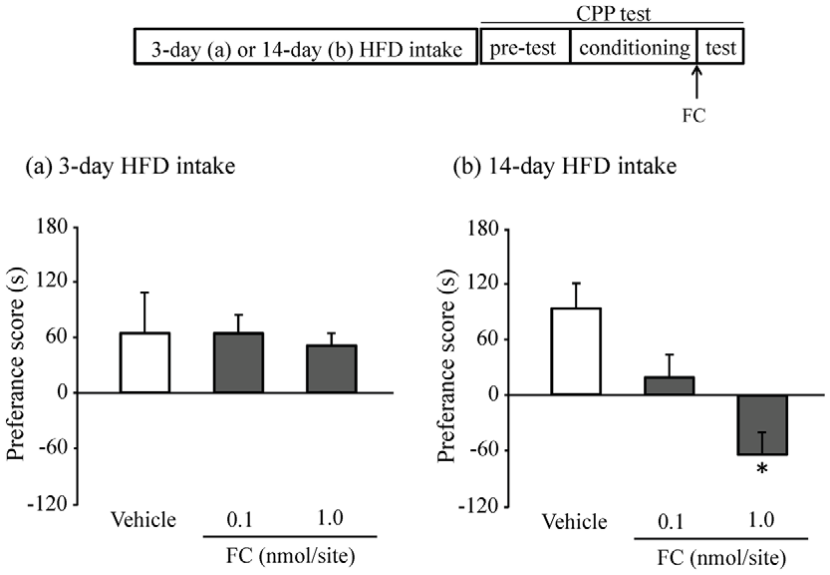
Effects of FC on preferences for a HFD. Results are expressed as the mean ± S.E.M. (a) The mice were fed a HFD for 3 days before the CPP test. (Vehicle, n = 10; 0.1 nmol/site, n = 10; 1.0 nmol/site, n = 11). (b) The mice were fed a HFD for 14 days before the CPP test. (Vehicle, n = 14; 0.1 nmol/site, n = 12; 1.0 nmol/site, n = 16). ^*^p<0.05 vs. Vehicle (Tukey-Kramer tests).

To evaluate HFD preferences, after HFD or SD consumption, the CPP test was conducted. Animals that had consumed a HFD for 1 day prior to CPP test, did not show a HFD preference. In contrast, animals that had consumed a HFD for 3 days prior to CPP conditioning showed a significant preference for the HFD compared with animals that had consumed a SD (group: F(1, 14) = 38.569, *p*<0.001, day: F(5, 70 = 1.172, *p* = 0.331, group x day: F(5, 70) = 1.153, *p* = 0.340, two-way ANOVA, ^*^ *p*<0.05, Student's t-test, Figure 1(a)). Next, we quantified the levels of hypothalamic 2-AG before and after the CPP test. At 3 and 7 days intake, the 2-AG levels after the test were significantly higher compared with HFD and SD intake group. The 2-AG levels after the test were significantly higher compared with before the test in the animals that had consumed a HFD for 3 and 7 days (3-day intake; F(3,20) = 9.620, 7-day intake; F(3,25) = 15.265, ^*^ *p*<0.05, Tukey-Kramer tests, Figure 1(b)). Moreover, animals that had consumed a HFD for 14, 28 and 42 days exhibited higher 2-AG levels before the test compared with animals that had not consumed a HFD, regardless of whether they had undergone the CPP test. The 2-AG levels after the test were significantly higher compared with HFD and SD intake group (14-day intake; F(3,24) = 14.566, 28-day intake; F(3,17) = 9.980, 42-day HFD intake; F(3,18) = 6.040, ^*^ *p*<0.05, Tukey-Kramer tests, Figure 1(b)). On the other hand, the 2-AG levels were not changed in SD intake group, regardless of whether they had undergone the CPP test (Figure 1(b)). The levels of astrocytes were examined to evaluate the effects of HFD consumption on the hypothalamus using the anti-GFAP antibody. Hypothalamic GFAP were increased. As determined by western blotting, the expression of GFAP was significantly increased after 14 days of HFD consumption, but not 3 days (^*^ *p*<0.05, Student's t-test, Figure 2(b)). The CB_1_ receptors antagonist O-2050 was used to examine the relationship between HFD preferences and the cannabinoid system. The administration of O-2050 (single injection, 10 mg/kg i.p.) before the CPP test suppressed HFD preferences in the animals that had consumed a HFD for 3 days (^*^ *p*<0.05, Student's t-test, Figure 3 (a)). The preference for a HFD in the animals that had consumed a HFD for 14 days was suppressed by the 14-day administration of O-2050 (14 times injection, one time/day, 10 mg/kg i.p.), but not administration of O-2050 before the CPP test (^*^ *p*<0.05, Student's t-test, Figure 3 (b)). Next, we tested whether O-2050 affected hypothalamic GFAP expression after 14 days of HFD consumption. The 14-day administration of O-2050 decreased the expression of GFAP in the hypothalamus (*†p*<0.05, Student's t-test, Figure 3(b)). Finally, we examined the direct relationship between hypothalamic GFAP expression and HFD preferences using FC. The administration of FC significantly decreased preferences for a HFD in the animals that had consumed a HFD for 14 days (F(2.31) = 9.885, ^*^ *p*<0.05, Tukey-Kramer tests, Figure 4(b)), but not in the animals that had consumed a HFD for 3 days (Figure 4(a)).

## Discussion

In this study, we found that there are two stages in the development of HFD preferences and the hypothalamic reward system is dynamically changed during the development of these preferences. During the first 3 days of HFD consumption, hypothalamic 2-AG was transiently increased, possibly by the rewarding effects of a HFD. Over 14 days of HFD consumption, 2-AG levels and the number of GFAP-IR astrocytes in hypothalamus were persistently increased. Taken together, we suggest that there is both an induction (over the first 3 days) and a maintenance phase (over the first 14 days) in the development of a HFD preference.

In this study, after 3 days of HFD consumption, the mice exhibited a HFD preference. On the other hand, SD fed mice did not show a HFD preference. Some studies indicate that HFD preference was developed in CPP test [1], [37]. However, in these studies, the conditioning was conducted more than 3 times. In this study, we conducted 2 times conditioning. Therefore, we concluded that 2 times HFD conditioning was enough to recognize the HFD paired-box, but did not develop the HFD preference. The CPP test was used widely to evaluate the several rewarding effects [1], [25], [26], [27], [28], [29]. However, it is difficult to examine the process of rewarding effects because mice were given HFD only during the conditioning period. As such, we used a previously described and modified CPP test [14], [15]. The mice were given the HFD or SD before the CPP test, and we assessed their preference by comparing the time spent in the HFD-paired side at baseline and following conditioning in each group (HFD consumption and SD consumption). Previously, we reported that this method is a valid and feasible way to evaluate HFD preferences continuously [14]. In this study, we examined the progress of a long-term HFD preference, for the first time.

In the animals that consumed the HFD for 3 or 7 days, hypothalamic 2-AG was significantly increased after the CPP test. However, there was no significant increment of 2-AG before the CPP test. Thus, the hypothalamic 2-AG may be increased by reinforcing effect of HFD. A recent study showed that levels of endocannabinoids in the hypothalamus were increased during hunger [20]. Levels of 2-AG have been shown to be regulated by leptin or glucose [38], [39]. In this study, mice were not deprived of food. There is the possibility that other factors regulate 2-AG levels when an animal has a HFD preference. Mizushige et al. reported that mRNA levels of proopiomelanocortin, a beta-endorphin precursor, are increased by the anticipation of consuming oil, and beta-endorphin is rapidly released after oil ingestion in oil-preferring rats [3], [4]. An increasing number of studies indicate that cannabinoids and opioids use partially overlapping mechanisms to modulate physiological processes, including reward and appetite [5], [18]. Thus, the high levels of 2-AG after the CPP test may be related to cannabinoid-opioid cross-talk. Further studies are needed to determine why 2-AG is increased transiently in the hypothalamus.

On the other hand, after 14 days of HFD consumption, hypothalamic 2-AG was significantly increased before the CPP test, and 2-AG levels were not changed after CPP test. And, the number of astrocytes was markedly increased in the hypothalamus. However, the number of neurons and microglia were not significantly changed by any duration of HFD consumption examined (data not shown). It is known that astrocytes are proliferated by cannabinoid-system [40]. Furthermore, cannabinoids are synthesized in astrocyte, not only in neurons [41]. Therefore, persistent increment of 2-AG may proliferate the hypothalamic astrocyte via cannabinoid-system.

To elucidate the relationship between the cannabinoid system and each stage of the development of HFD preferences, we examine the effects of the CB_1_ receptor antagonist, O-2050. The induction of a HFD preference (over 3 days) was attenuated by a single administration of O-2050 (before the CPP test). Accumulating studies show that CB_1_ receptor antagonists inhibit the acquisition of rewarding effect [13], [18], [19], [20], [21], [22]. In this study, our results suggest that activation of hypothalamic CB_1_ receptors by the transient increase in 2-AG levels during HFD conditioning is required for the induction of a HFD preference.

However, the HFD preferences that developed over 14 days of HFD consumption were attenuated by 14-day administration of O-2050, not but a single administration. Moreover, it should be noted that the expression of GFAP in the animals that consumed a HFD for 14 days was suppressed by 14-day administration of O-2050. It is known that astrocyte has CB_1_ receptors [41], [42]. The increment of 2-AG by HFD intake or rewarding effect might activate the hypothalamic astrocyte and developed HFD preference. Several studies show that astrocytes proliferate following cannabinoid stimulation and this is related to drug abuse and rewarding effects [33], [34], [35], [36], [40], [43]. Our results indicate that the development of a HFD preference in these animals required alteration of hypothalamic astrocytes to elicit long-term CB_1_ receptor activation. Thus, we suggest that an increase in hypothalamic astrocytes by 14 days of HFD consumption may be involved in the maintenance of a HFD preference.

Finally, we examined whether astrocytes mediate HFD preferences directly. In this regard, we previously reported that 1 nmol/site of FC can suppress astrocytic metabolism without neuronal damage [44]. We administrated the FC to dorsomedial hypothalamic nucleus (DMH), because astrocytes were activated in DMH. In this study, FC attenuated HFD preferences in the animals that consumed a HFD for 14 days, but not those that consumed a HFD for 3 days. In other words, FC suppressed HFD preference with activated astrocytes, and did not suppress HFD preference with non-activated astrocytes. Based on these results, we conclude that the maintenance of a HFD preference is mediated by GFAP-IR activated astrocytes via cannabinoid system. Thus, astrocyte proliferation and concomitant activation of CB_1_ receptors is directly involved in the development of HFD preferences over 14 days of HFD consumption. Accumulating data have shown that astrocytes are involved in many aspects of neuron-astrocyte interactions (e.g., the uptake and metabolism of glutamate, the dynamic sensing of neurotransmitters and neuromodulators, and the release of neuroactive factors) [31], [32], [45], [46], [47], [48], [49]. Recent studies suggest that astrocytes mediate rewarding effects including drug abuse via modulating glutamate [33], [34], [43]. Moreover, recent studies revealed that glutamatergic neurons and orexin in the hypothalamus activated the ventral tegmental area (VTA) [50], [51], [52]. Thus, activated astrocytes may activate hypothalamic orexin neurons via release of glutamate, and promote the rewarding processing. Additionally, astrocytes regulate the maturation of dendritic spines by direct contacts [53]. Our immunostaining results suggested that 14 days HFD intake induced the growth of astrocyte foot process. The astrocyte foot process may develop the maturation of dendritic spines to keep the HFD preference.

Based on our results (Fig. 1(b), Fig. 3 and Fig. 4), it is conceivable that the reward system's role in HFD preferences is different over 3 or 14 days of HFD consumption. Over the first 3 days, the induction of HFD preferences appears to be related to transient increases in hypothalamic 2-AG levels. Over the first 14 days, the maintenance of HFD preferences appears to be related to persistent increases in astrocyte levels and concomitant increases in CB_1_ receptor activation. The process of developing HFD preferences is still debatable, but our results indicate this process has both an induction and a maintenance phase. This is the first time that a multistage process of developing preferences for HFDs has been proposed.

## Methods and Materials

### Ethics Statement

All animal care and use procedures were performed in compliance with the regulations established by the Experimental Animal Care and Use Committee of Fukuoka University followed the Guidelines of the Science Council of Japan (approved no. 1007411 of institutional review board).

### Animals

Male ICR mice (25–35 g, Kyudo Experimental Animal Laboratory, Saga, Japan) were kept under a 12 h light/dark cycle (lights on from 07:00 to 19:00 h) in an air-conditioned room (23±2°C). They were fed a HFD (F2HFD1, Oriental Yeast Co, Tokyo, Japan) or a standard diet (SD) (CE-2, Clea Japan, Tokyo, Japan), and water was available *ad libitum*. The composition of each diet was as follows: fat, SD: 4.8 g/100 g (soybean-based), HFD: 5.3 g/100 g (butter-based); and calories, SD: 343.1 kcal/100 g, HFD: 414 kcal/100 g.

### Conditioned place preference (CPP) test

The CPP test was used to evaluate HFD preferences. Details of the CPP test were described previously [14], [15]. Mice were fed an SD or HFD before conducting the CPP test. Throughout the CPP test, mice ate about 3.4 g of the SD (nearly 10 kcal/day), which represents approximately 68% of the daily food consumption of mice fed *ab libitum* in similar housing conditions (about 14.7 kcal/day or 5.0 g of SD). A test chamber consisting of two sides of equal size (15×15×15 cm) was used for the CPP test. The walls of the dark side were black, and the light side was white. Both sides were equipped with a grid floor, and a hatched transparent sheet was placed on the grid of the dark side. The CPP test consisted of 3 stages. The pre-test was conducted on Day 1, the conditioning during Days 2–5, and the test on Day 6. In both the pre-test on Day 1 and the test on Day 6, each mouse was placed in the CPP apparatus and allowed free access to the light and dark sides in the absence of food for 15 min. The times spent in the light or dark side, respectively, were measured. During the conditioning on Days 2–5, each mouse was confined to the light or dark side for 30 min, respectively, in the presence of the SD or the HFD. Equal amounts of the SD or the HFD was offered to the mice during conditioning. Preference scores were expressed as the change in the time spent in the HFD-paired side before and after conditioning.

### Quantification of 2-AG

The levels of hypothalamic 2-AG were quantified by gas chromatography-mass spectrometry (GC-MS) as previously described [14], [54]. The tissues were isolated immediately after the CPP test. The hypothalamus was homogenized in 5 volumes of chloroform/methanol/Tris HCl buffer (50 mM, 2∶1∶1) containing butylated hydroxytoluene (BHT) (final, 0.05%) and 5 μg of URB602, which was added to block monoacylglycerol lipase activity. Homogenates were centrifuged at 20,400 g for 5 min (5°C). The aqueous phase and debris were collected and extracted twice with 1 volume of chloroform. The organic phases from the three extractions were pooled. The organic phase was dried under nitrogen before further purification. The organic phase was purified using Sep-Pak C-18 cartridges. The Sep-Pak C-18 cartridges were conditioned with 4 mL of methanol followed by a wash with 4 mL of water. Dried samples were diluted with 1.5 mL of acetone and 8 mL of water. After passing the sample, the cartridge was washed with 2 mL of acetone/water (20∶80), and the 2-AG was eluted with 5 mL of diethyl ether. The internal standard (IS), methyl heneicosanoate (50 ng), was added to 2-AG after C_18_ solid-phase extraction. The eluted solution was dried under nitrogen prior to derivatization. The 2-AG-containing fractions were derivatized by treatment with 30 μL of BSTFA at room temperature for 30 min. Derivatized samples were dried under nitrogen, resuspended in 20 μL of acetonitrile, and injected (splitless mode) into a GC-MS. A GCMS-QP2010 (Shimadzu, Kyoto, Japan) equipped with a AOC-20i autosampler (Shimadzu, Kyoto, Japan) was used for analysis in the positive electron ionization (EI) mode. GC-MS conditions were as follows: column, DB-5MS fused silica capillary column (30 m×0.25 mm i.d., 0.25 μm J&W scientific); column oven program, 10°C for 1 min followed by an increase to 290°C at 10°C/min. Injector temperature, 290°C; helium at a constant flow rate of 1.33 mL/min. The following ions were used for selected ion monitoring (SIM) analyses: 2-AG (*m/z* 522 for [M]^+^, *m/z* 507 for [M-methyl]^+^, *m/z* 432 for [M-TMSOH]^+^).

### Pharmacological effect of CB_1_ receptor antagonist, O-2050

O-2050 (TOCRIS, Ellisville, MO, USA) was dissolved in 1% Tween 80. O-2050 (10 mg/kg) and 1% Tween 80 (vehicle) were administered i.p. for 14 days (14-day) or 1 h before the Day 6 CPP test (test day).

### Immunostaining

Mice were perfused with saline and 4% paraformaldehyde. Their brains were cleared of fat and water using an auto degreasing unit (RH-12, Sakura Seiko, Tokyo, Japan) and then embedded in paraffin. Subsequently, 5-μm-thick sections were mounted on slides and dried at 37°C for 1 day. After deparaffinization and rehydration, sections were incubated in Tris-buffered saline (TBS) containing 0.1% Tween 20 (TBS-T) for 30 min. The sections were incubated with a blocking solution (3% goat serum) for 30 min at room temperature. Sections were then incubated overnight at 4°C with primary antibody (1∶500 rabbit glial fibrillary acid protein (GFAP)-antibody (Ventana AZ, USA) in blocking solution). The sections were stained using the avidin-biotin-peroxidase method. The sections were washed three times, incubated with goat anti-mouse or rabbit IgG for 1 h, washed again, and processed using the Vecstain ABC kit (Vector Labs, Burlingame, CA). They were immersed for 20–30 min in 0.05% diaminobenzidine, 0.05% H_2_O_2_, and imidazole buffer and then mounted on poly-L-lysine coated slides.

### Immunoblotting

The expression of GFAP protein was evaluated by western blotting following sample extraction by SDS-PAGE as described previously. Tissue samples were homogenized at 4°C for 1 min in lysis buffer with a protease inhibitor cocktail. Tissue extracts were centrifuged at 15,000 rpm at 4°C for 30 min. The same procedure was followed for the supernatant. SDS sample buffer was added to aliquots of tissue extracts containing 15 µg of total protein. Proteins were separated by SDS-PAGE (12–15% gel). Blotting was performed using a semi-dry method (BIORAD, Hercules, CA). The blots were blocked with 5% non-fat dry milk at 4°C, and incubated with anti-GFAP polyclonal antibodies (1∶200) in TBS-T, followed by goat anti-rabbit IgG AP conjugate (1∶1000) in TBS-T and bovine anti-goat IgG AP conjugate (1∶1000) in TBS-T. The blots were visualized by AP color reagents. The signal intensity of the blots was measured by an image analysis system (NIH Image, version 1.63).

### Pharmacological effects of FC

To test the hypothesis that the development of HFD preferences is directly related to astrocytes, we metabolically inhibited astrocytes using FC. The FC solution for intrahypothalamic injections was prepared by dissolving 8 mg of D, L-fluorocitric acid, Ba, salt (Sigma-Aldrich, St Louis, MO) in 1 ml of 0.1 mmol/L HCL. Two to three drops of 0.1 mmol/L Na_2_SO_4_ were added to precipitate the Ba^2+^. Two milliliters of 0.1 mmol/L Na_2_HPO_4_ was added, and the suspension was centrifuged at 1,000 g for 5 mins. The supernatant was diluted with 0.9% NaCl to the final concentration, and the pH was adjusted to 7.4. Stereotaxic surgery was carried out under sodium pentobarbital (50 mg/kg, i.p.; Tokyo Kasei, Tokyo Japan), and guide cannulas was stereotaxically implanted into DMH region (anterior: −1.70 mm from Bregma; lateral: 0.5 mm from Bregma; depth: 5 mm from the skull surface) before HFD intake. We administrated the FC according to our previously described methods and those of Paulsen *et al* [44], [55], [56]. The FC solution was microinjected into DMH 4 h before the CPP test. One microliter of the FC solution was injected continuously at a rate of 0.25 mL/min through a stainless steel cannula (28 gauge) connected to a 25-mL syringe driven by a slow-injection pump. At the completion of testing, brains were extracted and fixed in formalin. The brains were then sectioned with a sliding microtome, stained with cresyl violet, and mounted onto microscope slides for confirmation of injection sites. Miss injected mice were eliminated from the results.

### Statistical Analysis

Results are expressed as the mean ± the standard error of the mean (S.E.M.) Student's t-tests and Tukey-Kramer tests were used to determine statistically significant differences. Student's t-test were performed following two-way ANOVA for the CPP test. *P*<0.05 was considered to be statistically significant.
